# A methodological approach to identify external factors for indicator-based risk adjustment illustrated by a cataract surgery register

**DOI:** 10.1186/1472-6963-14-279

**Published:** 2014-06-25

**Authors:** Ursula Hahn, Irmingard Neuhann, Stefanie Schmickler, Frank Krummenauer

**Affiliations:** 1 Institut für Medizinische Biometrie und Epidemiologie, Fakultät für Gesundheit der Universität Witten/Herdecke, Alfred-Herrhausen-Straße 50, 58448 Witten, Germany; 2 OcuNet GmbH & Co KG, Friedrichstraße 47, 40217 Düsseldorf, Germany; 3 Medizinisches Versorgungszentrum Prof. Neuhann Augenheilkunde Neurologie Psychiatrie, Helene-Weber-Allee 19, 80637 München, Germany; 4 Augen-Zentrum-Nordwest Augenpraxis Ahaus, Domhof 15, 48683 Ahaus, Germany

**Keywords:** Quality assurance, Outcome quality, Confounder, External factors, Cataract surgery, Register

## Abstract

**Background:**

Risk adjustment is crucial for comparison of outcome in medical care. Knowledge of the external factors that impact measured outcome but that cannot be influenced by the physician is a prerequisite for this adjustment. To date, a universal and reproducible method for identification of the relevant external factors has not been published. The selection of external factors in current quality assurance programmes is mainly based on expert opinion. We propose and demonstrate a methodology for identification of external factors requiring risk adjustment of outcome indicators and we apply it to a cataract surgery register.

**Methods:**

Defined test criteria to determine the relevance for risk adjustment are “clinical relevance” and “statistical significance”. Clinical relevance of the association is presumed when observed success rates of the indicator in the presence and absence of the external factor exceed a pre-specified range of 10%. Statistical significance of the association between the external factor and outcome indicators is assessed by univariate stratification and multivariate logistic regression adjustment.

The cataract surgery register was set up as part of a German multi-centre register trial for out-patient cataract surgery in three high-volume surgical sites. A total of 14,924 patient follow-ups have been documented since 2005. Eight external factors potentially relevant for risk adjustment were related to the outcome indicators “refractive accuracy” and “visual rehabilitation” 2–5 weeks after surgery.

**Results:**

The clinical relevance criterion confirmed 2 (“refractive accuracy”) and 5 (“visual rehabilitation”) external factors. The significance criterion was verified in two ways. Univariate and multivariate analyses revealed almost identical external factors: 4 were related to “refractive accuracy” and 7 (6) to “visual rehabilitation”. Two (“refractive accuracy”) and 5 (“visual rehabilitation”) factors conformed to both criteria and were therefore relevant for risk adjustment.

**Conclusion:**

In a practical application, the proposed method to identify relevant external factors for risk adjustment for comparison of outcome in healthcare proved to be feasible and comprehensive. The method can also be adapted to other quality assurance programmes. However, the cut-off score for clinical relevance needs to be individually assessed when applying the proposed method to other indications or indicators.

## Background

Quality assurance procedures aim to highlight differences in the quality of medical care between providers. However, the outcome of medical care is not only determined by the medical contribution, but also by factors that cannot be influenced by the physician or medical care facility (referred to hereafter as “external factors”) [1-3]. A selective comparison of the contribution of medical care to the outcome measured by means of an indicator assumes that the effect based on these external factors will be negated by risk adjustment [1].

In the present study, a methodological approach based on two parallel test criteria is introduced to identify external factors requiring risk adjustment in quality assurance programmes. These criteria are “clinical relevance” and “statistical significance” of the relationship between the external factor and the measured outcome. Each criterion will be operationalised using statistical association analysis techniques.

The proposed method is demonstrated with data from a multi-centre register database on outcome quality in cataract surgery. The observed outcome indicators are “refractive accuracy” and “visual rehabilitation” 2–5 weeks after surgery. The external factors considered cover sociodemographic and quantitatively and qualitatively documentable patient characteristics.

## Methods

### Test criteria and their statistical parameterisation

The test criteria for identifying external factors requiring risk adjustment were derived according to published information on external factors in risk adjustment [4-6]. In the methodological approach presented here, a factor requires adjustment when it shows clinically relevant and statistically significant association with the outcome indicators being considered for a quality assurance programme.

For parameterisation of the clinical relevance criterion, the success rates (relative frequencies) for the indicators are determined in the presence and absence of each external factor. The absolute difference of these frequencies is interpreted as a measure of the factor’s clinical relevance for risk adjustment; if this difference exceeds a pre-specified cut-off, the relevance for risk adjustment is presumed to be demonstrated.

The criterion “statistical significance” is deemed to be met when demonstrated by at least one of two appropriate methods:

a) Univariate stratification: As a measure of the association between external factor and indicator, the Odds Ratio (OR) of the success rates under presence versus absence of the external factor can be determined. To correct for potentially differing frequencies of occurrence among documenting centres, the Odds Ratios should be estimated under stratification for centres, for example by means of the Mantel/Haenszel method. An OR < 1 then indicates that with the presence of the factor fewer patients achieve success in terms of the outcome indicator. To evaluate the statistical significance of the association, a local 99% confidence interval can be assigned to the OR estimate; if this local interval does not include “1”, the corresponding external factor is significantly associated with the outcome quality at the local 1% significance level.

b) Multiple adjustment: A multiple regression model can consider, in addition, the potential interaction of several external factors with each other. A (binary) outcome indicator can then be related to the set of external factors as potential explanatory variables by means of a multiple (logistic) regression model. In the regression model, the documenting centres can also be considered as explanatory variables; the association between external factor and outcome indicator can therefore be “adjusted” for centre differences instead of being stratified for centres as above. The multiple adjustment criterion of local statistical significance is met if the local 99% confidence interval of an external factor’s OR does not include “1”.

In general, the results of multiple regression modelling tend to be more informative than the results of univariate association analysis; on the other hand, the results of multiple regressions should only be applied if there is sufficient model fit (i.e. 80% in terms of an appropriate goodness of fit estimate). Therefore, it is generally recommended that univariate stratification should be applied in the first instance to obtain formal information on the significance of an external factor–outcome indicator association; additional multiple adjustment information should be considered if there is sufficient model fit, and this multiple adjustment criterion can then be considered as the more informative significance criterion.

Logistic regression models were constituted using backward selection based on local Likelihood Ratio tests at the nominal 1% level; the overall goodness of fit of a model was then estimated by means of Nagelkerke’s R^2^[2] coefficient. All analyses were performed using SPSS® (Version 20.0 for Windows®). Furthermore, results of the logistic regression modelling were validated using the public domain Software R.

### The MONIKA database

The OcuNet Group, an association of eye surgery practices in Germany, has been using the register survey “MONItoring in der ambulanten KAtaraktchirugie” [“MONItoring in outpatient CAtaract surgery”] — hereafter MONIKA — since 2005 as an internal quality assurance tool: the data are not publicly available. The database was originally set up as a quality monitoring instrument for the affiliated centres using regular reports. To meet the user’s demands in terms of readability an easy report systematic was chosen, endpoints and (possible) external factors were mostly quoted as binarized.

The database contains only completely anonymous data: each health care provider took responsibility to fully delete any patient information, which would have enabled identification of treated patients. After this process of data modification the anonymous site data were forwarded and gathered in an appropriate central data base. In summary, neither an approval from local independent ethics committees nor a permission from any other body or authority was required to constitute the project data contained in the MONIKA data set according to German Federal Data Protection Laws, e.g. as stipulated in the Berlin State Data Protection Law [7]. The documenting outpatient study centres are so-called “high-volume” centres with an above-average number of surgical procedures and a broad surgical range. The MONIKA database represents the clinical routine in the study centres. All patients with scheduled cataract surgery are eligible to be included in the database, regardless of diagnostic or therapeutic methods used. Anonymised patient characteristics, diagnostics, therapy, and type of implant (intraocular lens) are documented in the database.

Individual patient data are collected preoperatively, intraoperatively, and on three occasions postoperatively. Prior to surgery, personal characteristics like gender or age, visual acuity, axial length, intraocular pressure, target refraction, intraocular lens power, previous ocular surgery, presence of at least one surgically relevant ocular risk factor and presence of preexisting conditions, reducing visual acuity were documented. The intraoperative documentation covered the type of intraocular lens and surgical technique and possible complications during surgery. All cataract operations were performed by using phacoemulsification. On the first postoperative day as well as 2–5 weeks and three months later outcome characteristics including refraction, visual acuity and postoperative complications were recorded.

The following analyses will concentrate on documented outcome data assessed 2–5 weeks postoperatively. The first 100 consecutively documented records per centre were excluded to eliminate possible learning curve effects.

### Indicators of outcome quality

In microsurgery of age-related cataracts, the opaque crystalline lens is extracted via the so-called phacoemulsification technique (ultrasound) and replaced by an artificial intraocular lens in the capsular bag. The outcome quality of this surgery is usually measured using two outcome indicators: [8,9] “refractive accuracy” and “visual rehabilitation”.

“Refractive accuracy” describes the actual deviation of the preoperatively intended (target refraction (TR)) to postoperatively achieved refractive power of the eye (spherical equivalent (SE)). A “good” surgical outcome in terms of this indicator is defined as absolute TR – SE ≤ 0.5 dpt (corresponding to the fact that most patients with this outcome will not need correcting glasses for distance vision).

“Visual rehabilitation” describes the postoperative visual acuity. “Success” in terms of this indicator will be ascertained when the postoperative visual acuity cc (=cum correctione) is at least 1.0, corresponding to the physiological visual acuity of young adults.

### External factors considered

The external factors considered were categorised according to “sociodemographic” and “quantitatively” and “qualitatively” documented individual patient characteristics. Sociodemographic factors included “age at surgery > 80” and “female gender”; quantitatively documented external factors included “presence of a poor baseline visual acuity” (cc ≤ 0.1), “severe nearsightedness” (myopia, axial eye length ≥ 25 mm) and “severe farsightedness” (hyperopia; axial eye length ≤ 22 mm) [10]. Qualitatively documented external factors included the “presence of at least one pre-existing condition that (potentially) reduces the visual acuity” or “at least one known previous ocular surgery” or the “presence of at least one surgically relevant ocular risk factor”.

### Patient data

Between 2005 and 2011, five outpatient eye surgery centres of the OcuNet Group documented a total of 19,278 raw data sets (the first 100 consecutively documented data sets per centre were excluded) in the MONIKA database. However, the (voluntary and thus fluctuating) participation of the centres varied greatly over time: completeness of the MONIKA database varies according to postoperative examination, centre and observed indicators of the outcome quality. Most of the data for both the refractive and the visual outcome indicator were available for the examination 2–5 weeks after cataract surgery; the following analyses are therefore based on the data for this examination. Furthermore, to demonstrate the above methodological approach, only the data from three of the five documenting centres will be analysed, as two of the centres contributed relatively few valid data sets compared to the others.

A total of 14,924 patients were analysed (Table 1); 58.6% of these documented patients showed complete information for the postoperative outcome indicator “refractive accuracy” and 56.5% for the postoperative outcome indicator “visual rehabilitation” at 2–5 weeks.

**Table 1:** Absolute frequency of documented raw data sets per study centre over time for the consecutive documentation of cataract surgery in three centres

	**2005**	**2006**	**2007**	**2008**	**2009**	**2010**	**2011**	**total**
Centre 1	55	1,445	906	1,122	681	428	171	4,808
Centre 2			771	572	1,038	137		2,518
Centre 3		1,309	1,484	1,222	1,191	1,413	979	7,598
Total	55	2,754	3,161	2,916	2,910	1,978	1,150	14,924

## Results

Table 2 summarizes the reported prevalences per centre of the eight external factors with putatively relevant and significant association with the outcome indicators under consideration (thereby necessitating risk adjustment of success rates for these indicators). Note that for most external factors, the prevalences reported by centre 2 differ from those reported by centres 1 and 3, respectively. As a consequence, centre-stratified assessment of association estimates between these external factors and the outcome quality indicators was found to be required and reasonable.

**Table 2:** Reported prevalences [%] of sociodemographic, quantitatively and qualitatively documented external factors with putative relevance for two outcome indicators of cataract surgery (refractive accuracy and visual rehabilitation), stratified and cumulated for three outpatient centres

**Centre**	**1**	**2**	**3**	**1 – 3**
	**[%]**			
**Sociodemographic external factors**				
Age ≥ 80 years	28.1	18.7	22.4	23.6
Female gender	59.2	56.8	60.3	59.4
**Quantitatively documented external factors**				
Baseline visual acuity cc ≤ 0.1	14.4	9.2	7.0	9.8
Severe nearsightedness (myopia, axial eye length ≥ 25 mm)	7.5	11.0	8.7	8.7
Severe farsightedness (hyperopia, axial eye length ≤ 22 mm)	8.6	12.6	9.5	9.7
**Qualitatively documented external factors**				
Presence of at least one pre-existing condition (potentially) reducing visual acuity	40.8	27.6	32.7	35.4
Presence of at least one known previous ocular surgery	3.8	3.7	1.8	2.9
Presence of at least one surgically relevant ocular risk factor	28.1	11.0	26.2	24.9

The success rates without risk adjustment (for presence of all external factors) were 68.9% for refractive accuracy (centre range 62.0% to 74.1%) and 47.0% for visual rehabilitation (centre range 32.5% to 51.3%) for the combined data of the three centres. Table 3 summarises the success rate estimates [%] of the two indicators 2–5 weeks after cataract surgery under stratification for the presence of each respective external factor. With the exception of the factors “female gender” and “age at surgery > 80 years”, all “refractive accuracy” indicator-related success rates are lower in the presence of the respective factor than in its absence. For the indicator “visual rehabilitation”, all success rates were reduced in the presence of any external factor.

**Table 3:** Relative frequencies (“success rates” [%]) of two outcome indicators (visual rehabilitation and refractive accuracy) 2 – 5 weeks after cataract surgery, stratified for presence / absence of each putative external factor, based on the data of three outpatient centres

	**“Refractive accuracy”** │**SE** – **TR │ ≤ 0.5 dpt**			**“Visual rehabilitation”visual acuity cc ≥ 1.0**		
	**Absence**	**Presence**	**Difference**	**Absence**	**Presence**	**Difference**
	**[%]**	**[%]**	**Percentage points**	**[%]**	**[%]**	**Percentage points**
**Sociodemographic external factors**						
Age ≥ 80 years	67.1	69.4	- 2,3	51.6	33.1	18.5
Female gender	67.9	69.6	- 1,7	49.2	45.4	3.8
**Quantitatively documented external factors**						
Baseline visual acuity cc ≤ 0.1	70.0	59.6	10,4	48.6	34.3	14.3
Severe nearsightedness (myopia; axial eye length ≥ 25 mm) versus normal eye length (22.01 – 24.9 mm)	70.8	58.6	12,2	48.5	47.1	1.4
Severe farsightedness (hyperopia, axial eye length ≤ 22 mm) versus normal eye length (22.01 – 24.9 mm)	70.8	62.5	8,3	48.5	33.6	14.9
**Qualitatively documented external factors**						
Presence of at least one pre-existing condition (potentially) reducing visual acuity	69.5	67.6	1,9	48.5	33.6	14.9
Presence of at least one known previous ocular surgery	69.0	65.9	3,1	47.6	26.9	20.7
Presence of at least one surgically relevant ocular risk factor	70.0	65.6	4,4	49.0	41.1	7.9

*TR*: Target refraction, *SE*: Spherical equivalent 2–5 weeks after cataract surgery.

Visual acuity cc: best-corrected visual acuity 2–5 weeks after cataract surgery.

### Evaluation of the criterion “clinical relevance”

For refractive accuracy only two out of eight factors (“baseline visual acuity ≤ 0.1” and “severe myopia”) met the 10% difference criterion introduced here to characterise clinically relevant association between factors and outcome indicator. Whereas the external factor “severe nearsightedness” did not meet this criterion related to the indicator visual rehabilitation, “severe farsightedness” did as did most of the other external factors with outcome success rate reductions of up to 20.7%.

### Evaluation of the criterion “statistical significance” by stratification

The centre-stratified Odds Ratios resulting from stratified association analysis between the eight external factors and the respective outcome quality indicators are summarised in Table 4: locally significant associations (the 99% confidence interval of the respective OR does not include the value “1”) with the indicator visual rehabilitation were found for all external factors considered except “severe nearsightedness”. However, for refractive accuracy, only the quantitatively documented factors and the qualitative factor “presence of at least one surgically relevant risk factor” showed locally significant associations.

**Table 4:** Results of the univariate association analysis (under stratification for centres by means of the Mantel / Haenszel method) between putative external factors and two outcome indicators (refractive accuracy and visual rehabilitation) 2 – 5 weeks after cataract surgery, based on the combined data of three outpatient centres: Odds ratios (OR) and local 99% confidence intervals (CI)

	**“Refractive accuracy” **│**SE – TR │ ≤ 0.5 dpt**		**“Visual rehabilitation” visual acuity cc ≥ 1.0**	
	**OR**	**99% CI**	**OR**	**99% CI**
**Sociodemographic external factors**				
Age ≥ 80 versus age < 80	0.885	0.772–1.040	0.484	0.414–0.564
Female gender versus male gender	1.127	0.986–1.276	0.870	0.767–0.979
**Quantitatively documented external factors**				
Baseline visual acuity cc ≤ 0.1 versus > 0.1	0.613	0.492–0.777	0.556	0.443–0.688
Severe nearsightedness (myopia; axial eye length ≥ 25 mm) versus normal eye length (22.01 – 24.9 mm)	0.613	0.486–0.744	0.993	0.805–1.209
Severe farsightedness (hyperopia, axial eye length ≤ 22 mm) versus normal eye length (22.01 – 24.9 mm)	0.737	0.587–0.919	0.541	0.434–0.672
**Qualitatively documented external factors**				
Presence versus absence of at least one pre-existing condition (potentially) reducing visual acuity	0.915	0.804–1.047	0.508	0.442–0.571
Presence versus absence of at least one known previous ocular surgery	0.855	0.60–1.244	0.393	0.253–0.561
Presence versus absence of at least one surgically relevant ocular risk factor	0.773	0.675–0.888	0.732	0.632–0.840

*TR*: Target refraction, *SE*: Spherical equivalent 2–5 weeks after cataract surgery.

Visual acuity cc: best-corrected visual acuity 2–5 weeks after cataract surgery.

### Evaluation of the criterion “statistical significance” by centre-adjusted logistic regression

Logistic regression analysis (Table 5) showed an extremely low model fit (Nagelkerke’s adjusted R^2^ for refractive accuracy 3% and for visual rehabilitation 12%, respectively); as a consequence, the decision for risk adjustment for the external factors should not take these results into account and should be restricted to the univariate findings of stratified analysis. However, despite this low model fit, locally significant association at the 1% level was demonstrated for almost the same external factors as those already identified by stratification. In an exploratory sense, these regression results do not, in general, contradict the findings of the univariate association analysis, but cannot be called upon to confirm them.

**Table 5:** **Results of multiple logistic regression analyses to evaluate the association between putative external factors (explanatory variables) and two outcome indicators (refractive accuracy and visual rehabilitation) 2 – 5 weeks after cataract surgery based on the combined data of three outpatient centres: Odds ratios (OR) and local 99% confidence intervals (CI) for each explanatory variable; underlying model fit assessed by Nagelkerke’s adjusted R**^**2 **^**criterion for the respective multiple logistic regression models**

	**“Refractive accuracy”** │**SE – TR │ ≤ 0.5 dpt**		**“Visual rehabilitation” visual acuity cc ≥ 1.0**	
**Nagelkerke goodness of fit**	**0.03**		**0.12**	
	**OR**	**99% CI**	**OR**	**99% CI**
**Sociodemographic external factors**				
Age ≥ 80 years	0.872	0.749–1.030	0.478	0.409–0.560
Female gender	1.120	0.981–1.289	0.915	0.808–1.045
**Quantitatively documented external factors**				
Baseline visual acuity cc ≤ 0.1	0.705	0.567–0.891	0.568	0.433–0.698
Severe nearsightedness (myopia, axial eye length ≥ 25 mm)	0.612	0.498–0.760	0.958	0.771–1.200
Severe farsightedness (hyperopia, axial eye length ≤ 22 mm)	0.713	0.591–0.881	0.579	0.468–0.729
**Qualitatively documented external factors**				
Presence of at least one pre-existing condition (potentially) reducing visual acuity	1.016	0.878–1.178	0.519	0.449–0.584
Presence of at least one known previous ocular surgery	1.005	0.697–1.542	0.535	0.339–0.771
Presence of at least one surgically relevant ocular risk factor	0.766	0.660–0.896	0.834	0.730–0.962
**Centre contrasts**				
1 versus 3	0.763	0.644–0.892	1.304	1.110–1.503
2 versus 3	0.579	0.491–0.697	0.449	0.360–0.531

*TR*: Target refraction, *SE*: Spherical equivalent 2–5 weeks after cataract surgery.

Visual acuity cc: best-corrected visual acuity 2–5 weeks after cataract surgery.

## Discussion

In this paper, a method for identification of external factors relevant for risk adjustment using quantitative test criteria is proposed and demonstrated by using data from a multi-centre register database for outcome quality in cataract surgery. Risk adjustment serves to establish comparability of different medical facilities and is an important tool for quality assurance. While methodological risk adjustment procedures have already been adopted and are well established [11], little is known about how to identify the factors relevant for risk adjustment. Clinical relevance is discussed in some publications as a relevant characteristic in addition to statistical significance [5,6]. However, published literature does not include suggestions for the operationalisation of these criteria for specific indications and databases. In the present study, test criteria are qualitatively defined and parameterised using statistical methods for association analysis and by context-specific cut-off proposals.

Comprehensive analysis for identification of external factors relevant for risk adjustment should also consider the test criterion “uneven distribution among care providers”. We did not apply this criterion to the MONIKA database since the study centres are structurally similar (outpatient specialist providers) and, therefore, do not provide a true representation of the distortion in distribution of external factors throughout different levels of healthcare.

### Clinical relevance

An external factor has clinical relevance if patients are expected to experience disadvantages if it is present. As a measure of clinical relevance, the (absolute) deviation of the success rates with or without presence of the external factor was utilised in this analysis. Clinical relevance can be considered to be greater, the higher the deviation in success rates in the presence or absence of the external factor. In this investigation, taking into account the nature of the actual cataract surgery outcome indicator, a threshold limit value of at least 10 percent points was proposed to classify clinically relevant association between external factors and outcome quality indicators.

However, these cut-off points defining clinical relevance are based on normative settings and inherent reliability is not specified. Therefore, cut-off limits must be determined according to the actual indicators and their context in quality assurance procedures. For example, if an endpoint such as mortality is quantified by means of an indicator, a notably lower threshold limit value for the deviation in prevalences may be appropriate. An example for cataract surgery is the occurrence of postoperative endophthalmitis as (negative) indicator of the outcome quality, with a reported incidence between 0.5% and 0.015% [12]. Due to the high clinical relevance (high risk of blindness), a minor prevalence difference of predisposing external factors would be sufficient to declare clinical relevance for risk adjustment. In terms of this, utilisation of the above clinical relevance criterion based on the “absolute” prevalence difference must be critically discussed.

For positive endpoints like those choosen here determination of the cut-off value is a weighting and not justifiable by objective arguments: A cut-off point for clinical relevance of 5% rather than 10% would have resulted in two additional confirmed external factors (“severe farsightedness” and “presence of at least one surgically relevant ocular risk factor”). Lowering threshold means to consider more external factors and improve therefore the surrounding for a suitable quality comparison. The complexity of documentation, analysis, error rate and susceptibility to manipulation increase on the other hand.

A relative clinical relevance criterion would be another possible parameterization especially in studies with rare outcome variable. A relative risk scale might be considered in a study looking at relevant external factors responsible for rare events, e.g. endophthalmitis. However, in an interdisciplinary clinical setting an absolute excess risk scale is more acceptable. When being presented with an absolute difference most clinicians find such information easier to understand and more straight-forward to interpret as compared to a relative difference.

### Statistical significance

In the present study, statistical significance was deliberately parameterised by means of two established methodological approaches: first, univariate stratification association and second, multiple (logistic) regression analysis. Although both approaches allow for centre-corrected estimation of the association between each external factor and each outcome indicator, the univariate analysis of the association of external factors and the measured outcome quality did not allow simultaneous comparison of the influence of the surgical facility.

On the other hand, whereas logistic regression modelling enabled several external factors to be simultaneously related to one outcome indicator, model goodness of fit for this approach was crucial: although the univariate and the multiple approach identified the same external factors as those significantly associated with the refractive and visual outcome indicators, the extremely low model fit ≤ 12% for both models did not allow these modelling results to confirm the univariate approach in an analysis method perspective. The model fit might be acceptable with a (negative) endpoint occurring only rarely – e.g. complications after surgery – but not for binary, well confined positive endpoints. The type of variable – dichotomized instead of continuous - might have had negative impact on the model fit. Based on the results of this cataract example, we propose applying the univariate stratification approach in the first instance, despite its rather exploratory nature - and to consider the results from the regression modelling approach as additional information providing confirmation if there is sufficient model fit. It is the task of future studies and discussion to estimate to what extent (as measured by Nagelkerke’s R^2^) logistic regression modelling can be expected to explain the variation in the results of cataract surgery or other indications.

Both approaches must be discussed further with respect to multiple testing. Whereas both approaches accounted for centre heterogeneity by either stratification or adjustment for centres, both methods estimated eight external factor associations with each outcome quality indicator. As a consequence, a total of 2 × 8 associations were tested for significance, formally requiring multiplicity adjustment. However, regarding the exploratory nature of identifying external factors requiring risk adjustment in future analysis/reports, the above results should be considered in terms of locally significant association findings instead of formally correcting their confidence interval levels for multiplicity. Nevertheless, researchers should be aware of this “local” significance interpretation underlying the above significance criterion — and should reduce the number of external factors to be considered for risk adjustment.

### Factors relevant for risk adjustment

The selection of the external factors considered in this study is based on information in published literature on external factors demonstrating influence on success rates [9,13-21]. However, regardless of this discussion, risk adjustment in established quality assurance procedures for cataract surgery was carried out only for one indicator (visual rehabilitation) and for one factor (pre-existing conditions reducing visual acuity) [18,22-24]. The results of the analyses described here suggest that this limited risk adjustment does not sufficiently ensure a valid basis of comparison for the selective medical contribution to outcome quality in the context of quality assurance procedures.

The external factors considered in this study are categorized as sociodemographic and quantitatively measurable and qualitative patient-related characteristics documented on the basis of clinical findings. The objective of this categorisation was discovery of potential structural differences that can be attributed to the factor properties. As expected, the differences in prevalence between centres are smallest, the less complex and the more standardised the survey of a factor is. Identification of external factors based on qualitative clinical findings of varying complexity and standardisation therefore constitutes an intrinsic source of bias for appropriate risk adjustment for quality assurance procedures.

### Methodological considerations of the data quality of the MONIKA database

Adequate data quality is a prerequisite for transferability of the results from the MONIKA database to other quality assurance procedures. The time-shifted participation of the study centres could be regarded as a potential source of bias (Table 1). During the entire survey period there was no significant change in diagnostic or therapeutic methods employed, so that a systematic effect of the divergent entry period on the data quality seems unlikely. The incompleteness of the data could also be considered as a source for bias. However, reduced completeness has also been reported in other voluntary studies. The rates in the three centres of the MONIKA database are comparable with those of other publications with an equivalent study environment [25]. Lower response rates resulting from reduced commitment do not allow a conclusion on systematic bias [26].

### Internal and external consistency of the documentation

The internal consistency of the frequencies of quantitative to qualitative factors points to good data quality for the three study centres (Table 2). Generally, in these centres, a large proportion of elderly patients is associated with a large number of pre-existing conditions reducing visual acuity (and vice versa). In addition, the relationship of low baseline visual acuity and pre-existing conditions reducing visual acuity was described in all centre cohorts. Lastly, an association between refraction anomalies (“high myopia” or “high hyperopia”) and at least one previous ocular surgery or surgically relevant ocular risk factors is present in all centres.

Another approach for evaluation of the data quality is the comparison of the reported prevalence of external factors according to the MONIKA database (Table 3) with the literature. This analysis also confirms good documentation quality in the MONIKA database. The average age at surgery in the MONIKA database (73 years) is in the middle of the range of reported averages (67 years [22] to 76 years [23]). This also applies to the reported proportion of women (MONIKA: 59.4%; range of other publications 53% [27] to 66% [23]). The total prevalences documented in the MONIKA database for severe nearsightedness and severe farsightedness lie only slightly below those of a publication by a German university hospital [10].

Due to inconsistent definitions in the published literature, the comparison of prevalence of qualitative documented factors is more difficult. In the MONIKA database, the two factors “pre-existing conditions reducing final visual acuity” and “history of previous ocular surgery” are documented without quantification of the impact on attainable final visual acuity. In contrast, Murphy et al. use “ocular comorbidity that was expected to reduce postoperative acuity to 6/12 or worse” [22] as a definition and Jaycock et al. the limit “ocular copathology, identified as a reason for a guarded visual prognosis in the operated eye” [18]. As expected with this definition heterogeneity, the reported frequencies deviate strongly and range from 21.2% [22] to 41% [28], as compared to the cumulative reported prevalence for both factors reported in the MONIKA database of 36.1%.

In summary the data from the study centres show adequate consistency. The data quality can be considered sufficient for evaluation of the test criteria to identify relevant external factors for risk adjustment of outcome indicators in cataract surgery.

## Conclusion

In this study, a method is presented for identifying external factors, which is to be used as part of a quality assurance procedure for outcome indicator-dependent risk adjustment. Relevance for risk adjustment is proposed if the factor’s association with an outcome quality indicator is found to be clinically relevant and statistically significant. Clinical relevance was assumed if the indicator success rate deviates more than 10 percentage points with or without adjustment. The method proved to be feasible and comprehensible when applied to a multicentre register database on outcome quality in cataract surgery. External factors with indicator-dependent relevance for risk adjustment could be determined. The method can be adapted to other quality assurance programms, it might also be useful to identify unknown risk factors. However, the cut-off score for clinical relevance needs to be individually assessed when applying the proposed method to other indications or indicators.

## Abbreviations

OR: Odds ratio; SE: Spherical equivalent; TR: Target refraction; Visus cc: visus cum correctione.

## Competing interests

None of the authors has financial; political or otherwise competing interests in the content and results presented in this manuscript; no external funding was received for this investigation. Parts of the data contained in this manuscript have been presented at the 2012 annual meeting of the German Association of Ophthalmologists (DOG Congress).

## Authors' contributions

UH coordinated the MONIKA register trial underlying this methodological investigation and participated in this investigation’s design, developed the issue of the manuscript and drafted its first version. She participated in performing the statistical analysis of the presented date; furthermore she thoroughly revised the further manuscript version by bringing together the respective co-author perspectives and intergrating the reviewers’ comments and suggestions. IN contributed data to the MONIKA database underlying this methodological investigation and critically revised this manuscript draft by bringing in ophthalmological expertise. StS was principal clinical investigator for the MONIKA register underlying this methodological investigation and contributed a major part of its clinical patient data; furthermore she revised this manuscript draft by bringing in the trial site perspective. FK was methodological co-designer of the MONIKA register by bringing in its statistical design and analysis; he further designed the statistical evaluation concept proposed in this methodological presentation and coordinated the data analysis and its result representation. Furthermore he thorouhly revised the first manuscript draft from the methodological perspective and contributed major parts for its revision. All authors read and approved the final manuscript.

## Pre-publication history

The pre-publication history for this paper can be accessed here:

http://www.biomedcentral.com/1472-6963/14/279/prepub

## References

[B1] Donabedian A Evaluating the quality of medical care Milbank Mem Fund Q 1966 14 3 166 206 5338568

[B2] Mant J Process versus outcome indicators in the assessment of quality of health care Int J Qual Health Care 2001 14 6 475 480 11769750 10.1093/intqhc/13.6.475

[B3] Veit C Hertle D Pay-for-Performance in Health Care System: Lessons learned and steps forward Am Inst contempory Ger Studies Policy Report 2012 14 28ff

[B4] Reiter A Fischer B Kötting J Geraedts M Jäckel WH Döbler K QUALIFY: Ein Instrument zur Bewertung von Qualitätsindikatoren Z Evid Fortbild Qual Gesundh wesen (ZEFQ) 2008 14 683 688 10.1016/j.zgesun.2007.11.003 18309894

[B5] Sakallaris B Jastremski C Von Rueden K Clinical decision support systems for outcome measurement and management AACN Clin Issues 2000 14 3 351 362 11276650 10.1097/00044067-200008000-00003

[B6] Heller G Zur Messung und Darstellung von medizinischer Ergebnisqualität mit administrativen Routinedaten in Deutschland Bundesgesundheitsblatt Gesundheitsforschung Gesundheitsschutz 2008 14 10 1173 1182 18985411 10.1007/s00103-008-0652-0

[B7] Berliner V Gesetz zum Schutz personenbezogener Daten in der Berliner Verwaltung (BlnDSG) Gesetzes und Verordungsblatt Berlin 2007 14 11 598

[B8] Schein O Steinberg E Javitt J Cassard S Tielsch J Steinwachs D Legro M Diener-West M Sommer A Variation in cataract surgery practice and clinical outcomes Ophthalmology 1994 14 6 1142 1152 8008356 10.1016/s0161-6420(94)31209-7

[B9] American Academy of Ophthalmology Cataract and Anterior Segment Panel Preferred Practice Pattern® Guidelines. Cataract in the Adult Eye 2011 San Francisco, CA: American Academy of Ophthalmology Available at http://www.aao.org/ppp

[B10] Haigis W Ashok G, Lin JT IOL calculations in long and short eyes Mastering Intraocular Lenses (Principles, Techniques & Innovations) 2007 92 98

[B11] Selbmann H Warncke W Eissner H Comparison of hospitals supporting quality assurance Methods Inf Med 1982 14 2 75 80 6808319

[B12] Barry P Seal DV Gettinby G Lees F Peterson M Revie CW ESCRS study of prophylaxis of postoperative endophthalmitis after cataract surgery: Preliminary report of principal results from a European multicenter study J Cataract Refract Surg 2006 14 3 407 410 16631047 10.1016/j.jcrs.2006.02.021

[B13] de Juan V Martin R Perez I Herreras JM Influence of axial length in refractive outcome after cataract surgery Arch Soc Esp Oftalmol 2010 14 4 144 148 20858402

[B14] Kugelberg M Lundstrom M Factors related to the degree of success in achieving target refraction in cataract surgery: Swedish National Cataract Register study J Cataract Refract Surg 2008 14 11 1935 1939 19006741 10.1016/j.jcrs.2008.06.036

[B15] Schein O Steinberg E Cassard S Tielsch J Javitt J Sommer A Predictors of outcome in patients who underwent cataract surgery Ophthalmology 1995 14 5 817 823 7777281 10.1016/s0161-6420(95)30952-9

[B16] Desai P Minassian D Reidy A National cataract surgery survey 1997–8: a report of the results of the clinical outcomes Br J Ophthalmol 1999 14 12 1336 1340 10574810 10.1136/bjo.83.12.1336 PMC1722910

[B17] Lundstrom M Stenevi U Thorburn W Cataract surgery in the very elderly J Cataract Refract Surg 2000 14 3 408 414 10713238 10.1016/s0886-3350(99)00418-6

[B18] Jaycock P Johnston R Taylor H Adams M Tole D Galloway P Canning C Sparrow J The Cataract National Dataset electronic multi-centre audit of 55,567 operations: updating benchmark standards of care in the United Kingdom and internationally Eye (Lond) 2009 14 1 38 49 18034196 10.1038/sj.eye.6703015

[B19] Lundstrom M Barry P Leite E Seward H Stenevi U 1998 European Cataract Outcome Study: report from the European Cataract Outcome Study Group J Cataract Refract Surg 2001 14 8 1176 1184 11524187 10.1016/s0886-3350(01)00772-6

[B20] Norregaard J Bernth-Petersen P Alonso J Andersen T Anderson G Visual functional outcomes of cataract surgery in the United States, Canada, Denmark, and Spain: report of the International Cataract Surgery Outcomes Study J Cataract Refract Surg 2003 14 11 2135 2142 14670422 10.1016/s0886-3350(03)00340-7

[B21] The Royal College of Ophthalmologists Cataract Surgery Guidelines 2010 2010 London: The Royal College of Ophthalmologists

[B22] Murphy C Tuft S Minassian D Refractive error and visual outcome after cataract extraction J Cataract Refract Surg 2002 14 1 62 66 11777711 10.1016/s0886-3350(01)01027-6

[B23] Lundstrom M Stenevi U Thorburn W The Swedish National Cataract Register: A 9-year review Acta Ophthalmol Scand 2002 14 3 248 257 12059861 10.1034/j.1600-0420.2002.800304.x

[B24] Lundstrom M Barry P Henry Y Rosen P Stenevi U Evidence-based guidelines for cataract surgery: Guidelines based on data in the European Registry of Quality Outcomes for Cataract and Refractive Surgery database J Cataract Refract Surg 2012 14 6 1086 1093 22541829 10.1016/j.jcrs.2012.03.006

[B25] Gale R Saldana M Johnston R Zuberbuhler B McKibbin M Benchmark standards for refractive outcomes after NHS cataract surgery Eye (Lond) 2009 14 1 149 152 17721503 10.1038/sj.eye.6702954

[B26] Hahn U Krummenauer F Neuhann I Ergebnisbezogene Erfolgsraten der Kataraktoperation - Ergebnisse einer systematischen Literaturübersicht DER OPHTHALMOLOGE 2012 14 575 582 22534745 10.1007/s00347-012-2577-0

[B27] Zaidi FH Corbett MC Burton BJ Bloom PA Raising the benchmark for the 21st century–the 1000 cataract operations audit and survey: outcomes, consultant-supervised training and sourcing NHS choice Br J Ophthalmol 2007 14 6 731 736 17050577 10.1136/bjo.2006.104216 PMC1955623

[B28] Baranano AE Wu J Mazhar K Azen SP Varma R Visual acuity outcomes after cataract extraction in adult latinos. The Los Angeles Latino Eye Study Ophthalmology 2008 14 5 815 821 17826836 10.1016/j.ophtha.2007.05.052 PMC4864722

